# The Effect of Sensor Feature Inputs on Joint Angle Prediction across Simple Movements

**DOI:** 10.3390/s24113657

**Published:** 2024-06-05

**Authors:** David Hollinger, Mark C. Schall, Howard Chen, Michael Zabala

**Affiliations:** 1 Department of Mechanical Engineering, Auburn University, Auburn, AL 36849, USA; dzh0063@auburn.edu (D.H.); zabalme@auburn.edu (M.Z.); 2 Department of Industrial & Systems Engineering, Auburn University, Auburn, AL 36849, USA; 3 Department of Industrial & Systems Engineering and Engineering Management, University of Alabama-Huntsville, Huntsville, AL 35899, USA; hc0060@uah.edu

**Keywords:** wearable sensors, accelerometers, gyroscopes, movement intent prediction

## Abstract

The use of wearable sensors, such as inertial measurement units (IMUs), and machine learning for human intent recognition in health-related areas has grown considerably. However, there is limited research exploring how IMU quantity and placement affect human movement intent prediction (HMIP) at the joint level. The objective of this study was to analyze various combinations of IMU input signals to maximize the machine learning prediction accuracy for multiple simple movements. We trained a Random Forest algorithm to predict future joint angles across these movements using various sensor features. We hypothesized that joint angle prediction accuracy would increase with the addition of IMUs attached to adjacent body segments and that non-adjacent IMUs would not increase the prediction accuracy. The results indicated that the addition of adjacent IMUs to current joint angle inputs did not significantly increase the prediction accuracy (RMSE of 1.92° vs. 3.32° at the ankle, 8.78° vs. 12.54° at the knee, and 5.48° vs. 9.67° at the hip). Additionally, including non-adjacent IMUs did not increase the prediction accuracy (RMSE of 5.35° vs. 5.55° at the ankle, 20.29° vs. 20.71° at the knee, and 14.86° vs. 13.55° at the hip). These results demonstrated how future joint angle prediction during simple movements did not improve with the addition of IMUs alongside current joint angle inputs.

## 1. Introduction

The use of machine learning for human intent recognition in health-related applications, such as exercise monitoring, human–robotic assistance, and rehabilitation, has grown considerably in recent years [1]. Human movement intent prediction (HMIP), such as future joint angle prediction, is a challenging task, where information of user activity is utilized to determine or estimate future body motion. Oftentimes, HMIP incorporates information about user activity in the form of time-series signals from wearable sensors to approximate limb motion 100 ms into the future [2]. HMIP has applications for human–robot interaction, exoskeleton control, and rehabilitation [3,4]. Determining sensor combinations that optimize HMIP, however, poses a grand challenge in the field of biomechanics [5,6,7,8,9]. There are several factors that influence joint angle prediction from wearable sensors, such as the number of sensors, the types of sensors, and the placement of sensors [10]. Although multiple sensors may be needed to characterize human motion, too many sensors may burden the user, increase the setup time, and require additional computation [10]. Furthermore, an excessive number of physical sensors could alter the user’s natural movement, resulting in movement strategies that differ from their ordinary daily lives.

Wearable sensors, including inertial measurement units (IMUs), provide a portable means of accessing user motion outside the confines of a lab [11,12]. IMUs are widely used to estimate joint, segment, and muscle kinematics and kinetics from accelerations, angular velocities, and local magnetic fields [10,13]. Although IMUs offer a popular and modular solution to HMIP, excessive computational complexity due to additional sensors may delay intent prediction. This can be problematic for real-time exoskeleton applications since exoskeletons must react faster than the onset of physiological reactive joint moments of about 130 ms [14]. Furthermore, real-time controllers of assistive devices have been shown to reduce in functional performance as delays exceed 125 ms and be considered unacceptable for real-time applications with delays above 300 ms [15,16]. Including additional sensors may increase the computational complexity, which may increase the runtime for predicting user intent. Conversely, too few sensors may inadequately capture the user’s intent during complex tasks or task transitions [17,18].

Along with sensor count and type, precise sensor location for extracting relevant physiological signals is essential for effective HMIP [19]. For example, sensors placed on the foot and shank can adequately measure ankle kinematics (r^2^ of 0.97 for ankle angle) [20]. Sensors are often positioned proximal and distal to the neighboring joint of interest (e.g., thigh–shank for knee kinematics and thigh–waist for hip kinematics) [20,21]. For example, Molinaro et al. (2020) estimated sagittal plane hip torque using data from rotary encoders mounted on the sagittal plane of the hip and bilateral thigh IMUs (3D accelerometer and gyroscope) [21]. Additionally, Dey et al. (2020) used thigh kinematics (angles, angular velocity, and angular acceleration) as inputs to a Random Forest regression to estimate joint angles and moments at the ankle and knee [20].

Previous work using machine learning algorithms from wearable sensors has shown promise for accurate HMIP. For example, Hollinger et al. (2023) used a Random Forest, long short-term memory (LSTM), and bidirectional LSTM to predict lower-limb joint angles 100 ms into the future using EMG, IMU, and joint kinematic inputs. Although the bidirectional LSTM resulted in a mean root mean squared error (RMSE) of 1.42–5.71°, the study was limited to level-ground walking and did not explore feature combinations to reduce the number of input signals [22]. Kazemimoghadam and Fey (2021) examined the effect of training task combinations for task recognition accuracy of transitions and found that including data of the target task in the training data enhanced the prediction accuracy on unanticipated locomotion tasks [17]. Mundt et al. (2020) used an artificial neural network using IMU sensors to predict joint angles with a mean RMSE < 4.8° [23]. Xiong et al. (2019) used an artificial neural network to predict joint angles and moments using EMG and joint angle input features and reported a lower-limb joint moment normalized RMSE (NRMSE) < 7.89% [24]. Yun et al. (2014) used a statistical stochastic approach called Gaussian process regression with anthropometry (14 body parameters) as input features to predict joint motions with a mean error < 4.3° at the ankle [25]. Farmer et al. (2014) used a nonlinear autoregressive model with EMG and kinematic inputs to estimate the future prosthetic ankle angle in three transtibial amputees [26]. The prior work mentioned here clearly shows how machine learning can accurately predict joint angles. However, what remains unknown is the optimal set of feature combinations from wearable sensors that would result in the greatest prediction accuracy while simultaneously limiting the number of attached sensors for the user. 

Oftentimes, it is preferred to use a subset of sensor inputs if the reduced number of inputs provides a similar performance. For example, joint angle inputs from the leading or trailing limb have been reported to result in the greatest prediction accuracy and did not improve with the addition of gyroscope and acceleration signals for predicting level-ground straight walking, turns, and transitioning from walking to stair ascent [18]. Certain clinical applications may also have limited time and resources and could benefit from using a sensor configuration that can be easily collected in the clinic [27]. Bao and Intille (2004) found that two accelerometers placed on the wrist and thigh resulted in comparable accuracy to five accelerometers placed on the hip, wrist, arm, ankle, and thigh for classifying twenty everyday tasks [28]. Therefore, HMIP is often a balance between achieving high-level accuracy while simultaneously minimizing the number of sensors worn to maximize practical adoption.

A hybrid approach using a combination of subject-independent and subject-dependent training data has also shown promise for lower-limb joint-level prediction [29]. Combining subject-dependent (individual-specific) and subject-independent (inter-individual) data from shank, knee, and hip kinematics and anthropometry as inputs for predicting ankle torque also highlights how machine learning algorithm accuracy is improved by the inclusion of multiple participants’ data. Dey et al. (2021) found that incorporating personalized data for training a machine learning algorithm resulted in the best accuracy compared to the baseline user-independent model [29]. However, Dey et al. (2021) did not examine the feature combinations of the input data, which could offer more granular-level information for HMIP. Kazemimoghadam and Fey (2021) observed signals of the lower limb and torso but did not compare the combinations of input signals to optimize prediction accuracy [17]. They did, however, recommend future studies to identify top input signals contributing to accurate prediction of the user’s intended tasks. Despite a plethora of research studies dedicated to using machine learning for HMIP, it is still unclear if the number and location of sensors optimize the prediction accuracy. Therefore, the objective of this study was to analyze various combinations of input signals that maximize the machine learning prediction accuracy of joint angles for multiple actions, focusing on simple movement tasks, such as knee flexion, knee extension, ankle plantarflexion, ankle dorsiflexion, hip flexion, and hip extension, while minimizing the required number of sensors to do so. In this study, we trained and evaluated a Random Forest algorithm to predict user intent 100 ms into the future across simple movement tasks from various combinations of sensor feature inputs. Unlike prior studies, which largely focused on prediction accuracy with various algorithms, this study seeks to determine the optimal set of feature combinations from wearable sensors to maximize the prediction accuracy while minimizing the number of sensors. This study begins with simple movements as a starting point and differs from studies that only analyze complex tasks. This approach allows for a more controlled evaluation of how sensor number and location affect joint angle prediction. Although deep learning models, such as LSTM and CNN, have been shown to produce excellent prediction accuracy for HMIP, their complexity makes it challenging to interpret results and use them for real-time applications where low latency is crucial [30,31,32]. Therefore, we decided to use Random Forest for HMIP because it has an excellent tradeoff of speed, accuracy, interpretability, and simplicity [32,33]. 

Determining IMU quantity and placement that accurately predict joint angles has several practical applications for improving functional outcomes during rehabilitation. Specifically, stroke survivors may use joint angle prediction for personalized rehabilitation protocols for enhancing healthy gait biomechanics [34]. Athletes recovering from sports injuries can also benefit from accurate joint angle prediction of their movements to detect the risk of future injury from compensatory movement strategies [35]. Finally, since hip fractures were reported in 1–2% of falls among the elderly population, accurate joint angle prediction can potentially improve exercise protocols that enhance mobility and mitigate the risk of falling [36].

As mentioned by Chen et al. (2023), sensor fusion methods based on static and quasi-static conditions are still needed for estimating joint angles [37]. This study, therefore, focused on simplistic movements to assess the initial capabilities of wearable sensors for HMIP under quasi-static conditions. We aimed to start with these basic motions to lay the foundation for the system’s performance before estimating joint angles in more complex scenarios, such as walking, jogging, and stair climbing. We hypothesized that the prediction accuracy of ankle, knee, and hip angles across six isolated movements would increase with the addition of IMUs attached to adjacent body segments. We also hypothesized that the addition of IMUs attached to non-adjacent body segments would decrease the prediction accuracy.

## 2. Materials and Methods

**Figure 1:**
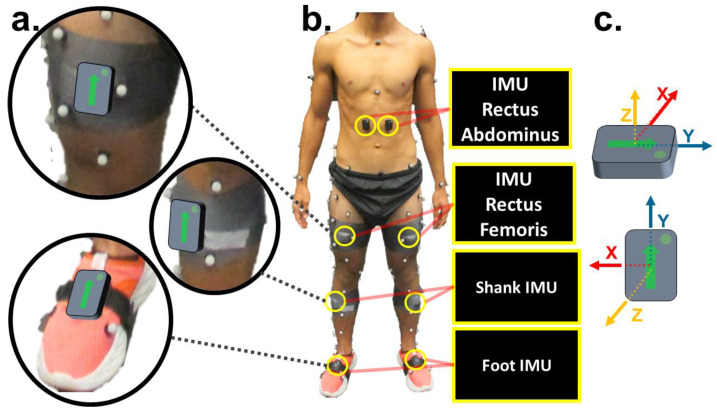
Experimental setup. (**a**) IMU placement of the lower limb and foot. (**b**) Frontal view of the placement of retroreflective markers and IMU sensors. (**c**) IMU sensor axes of flexion/extension, abduction/adduction, and internal/external rotation following the x-z-y coordinate system.

Twenty-eight participants (16 males, 12 females, aged 21.96 ± 2.83 years; height 1.73 ± 0.10 m; weight 71.80 ± 11.86 kg) provided written informed consent to volunteer in the study at the Auburn University Biomechanical Engineering (AUBE) Laboratory. The test protocol was approved by the Auburn University IRB (protocol no. 17-279-MR 1707) and followed the same experimental protocol as Hollinger et al. (2023) [22]. However, in this study, we analyzed eight IMUs (Trigno IM Sensors, version 3.5, Delsys Inc., Boston, MA, USA) attached bilaterally to segments of the torso, thigh, shank, and dorsal aspect of the foot (Figure 1). The IMU sensors recorded three-dimensional acceleration and angular velocity sampled at 148 Hz. Delsys Trigno IM Sensors were chosen because they provide reliable measurements of triaxial accelerometry and angular velocity, which are essential for accurate joint angle prediction. Since these sensors are commonly used in industry, clinical, and academic research, they improved the overall comparability of our study with existing literature and ensured reproducibility for future studies.

**Figure 2:**
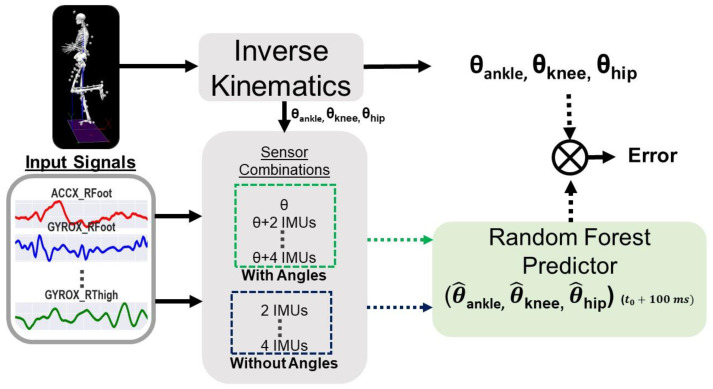
Lab-based data analysis and algorithm overview. Trials of multiple actions (action-agnostic) were trained with sensory information input for a continuous Random Forest predictor. Joint angle predictions were compared to angles obtained from motion capture using inverse kinematics.

Synchronization between motion capture and IMU sensors was performed similar to the methodology explained by Hollinger et al. (2023) [22]. The overall testing workflow is shown in Figure 2, where joint angles derived from motion capture were treated as ground truth values and compared to joint angle predictions (Figure 2). The Random Forest predictor was fed 50 ms of input data to forecast joint angles 100–150 ms later. Following a prediction, the input data shifted in increments of 50 ms and the prediction process was repeated until the end of the movement trial. Random Forest was chosen for its high estimation accuracy and robustness to noise and outliers, which make it ideal for handling nonlinear relationships between inertial data and joint angles [38]. Since Random Forest is an ensemble of decision trees with efficient parallelization, the model is computationally efficient for making quick predictions [38]. This aspect is an important consideration for real-time applications where fast predictions are essential. Participants performed three repetitions of six joint-level movements. The movements were ankle plantarflexion, ankle dorsiflexion, knee flexion, knee extension, hip flexion, and hip extension. We performed subject-dependent training and testing by randomly selecting a single limb and two repetitions of a simple movement for training the Random Forest predictor. The predictor then performed testing on the third trial, which was excluded from the training set of the same participant. For 28 participants and 6 simple movements, this approach resulted in 168 trained models. The Random Forest hyperparameters were chosen from default values provided by Python’s scikit-learn library version 1.4.2 (n_estimators = 100, criterion = ‘squared_error’0, max_depth = none, min_samples_split = 2, and min_samples_leaf = 1). The default settings were chosen so we could analyze the most simplistic scenario of a Random Forest configuration without the influence of hyperparameter tuning.

**Figure 3:**
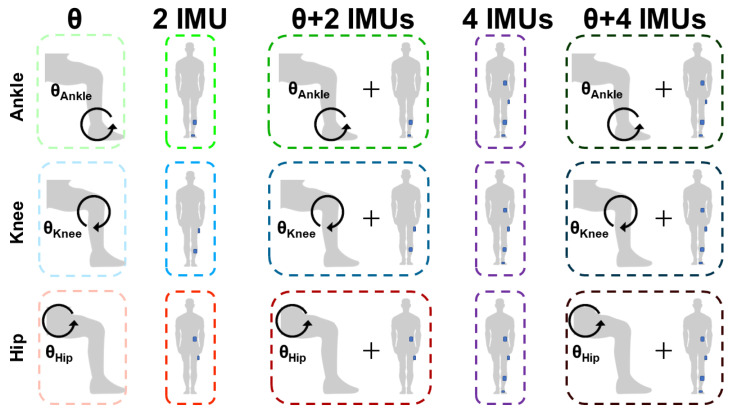
Input signal combinations for predicting ankle, knee, and hip angles. The blue rectangles on the person represent the evaluated IMU sensor configuration. Two IMUs were evaluated to the neighboring joint as a reduced sensor set. The two IMU sensors were located at the shank and foot to predict ankle angles, the thigh and the shank to predict knee angles, and the torso and thigh to predict hip angles. An identical set of four IMU sensors was evaluated at the foot, shank, thigh, and torso to predict ankle, knee, and hip angles.

Sensor features were systematically selected as inputs to the Random Forest algorithm. The selected features varied by number and type. The number of sensors varied to include a full set of IMUs on the ipsilateral single side of the joint (four IMUs) versus a reduced set of IMUs (two IMUs; Figure 3). Since we randomly tested on a single side for joint angle prediction, the maximum sensor configuration consisted of four IMUs. The rationale for comparing a full set of IMUs on the ipsilateral side to a reduced set of IMUs was to strategically compare sensors per body segment with one sensor per adjacent body segment. Although the addition of IMUs attached to non-adjacent segments did not exhibit the same amount of motion as IMUs attached to adjacent segments neighboring the joint, some movement still occurred. For instance, the foot IMU moves as the user lifts their foot in the air during knee extension and flexion and hip extension and flexion. Therefore, even though it may seem as though non-adjacent IMUs did not provide any relevance for predicting joint angles, information may potentially be lost by ignoring the IMUs attached to these segments.

The types of features in each feature set were also varied based on three levels: joint angles (θ), IMU, and IMU + θ. The overall number of feature inputs ranged from 1 input (joint angles, θ) to 25 input signals comprised of 4 IMU sensors (Acceleration X, Acceleration Y, Acceleration Z, Angular Velocity X, Angular Velocity Y, and Angular Velocity Z per IMU sensor) and joint angles (θ). Although obtaining joint angles from optical motion capture is not practical for real-time applications, the data could be derived through an onboard potentiometer or rotary encoder. RMSE was the chosen evaluation metric for prediction performance because the metric penalizes outliers more heavily compared to the mean absolute error (MAE). This is because RMSE is an L_2_ norm of the error vector measured between the predicted and the true values. A lower RMSE means the predictor is more accurate compared to a higher RMSE, as shown in the following equation:

(1) $$RMSE=\sqrt{\frac{\sum_{i=1}^{N}{\left(Y\left(i\right)-\hat{Y}\right)}^{2}}{N}}$$

where N represents the number of predictions, $\hat{Y}$ represents the predicted angle, and $Y\left(i\right)$ represents the measured angle at value i. 

Cohen’s *d* was calculated to evaluate the effect size among pairwise comparisons of the sensor combinations and was analyzed for small (*d* = 0.2), medium (*d* = 0.5), and large (*d* ≥ 0.8) effects [39]. Bland–Altman plots with Pearson correlation coefficients were implemented to assess the strength of the linear relationship between the differences between the predicted and measured joint angles and the measured joint angle, as shown in the following equation:

(2) $$r=\frac{\sum \left(x-m_{x})(y-m_{y}\right)}{\sqrt{\sum {\left(x-m_{x}\right)}^{2}\sum {\left(y-m_{y}\right)}^{2}}}\;$$

where x and y are two vectors of length N, and $m_{x}$ and $m_{y}$ are the means of x and y.

The plots were set with upper and lower bounds capturing a 95% level of agreement. To test our hypotheses, we performed a one-way analysis of variance (ANOVA) to compare the various sensor inputs for predicting the joint angles shown in Figure 3. Significant ANOVA tests prompted post hoc Tukey’s honest significant difference (HSD) tests. The Tukey–Kramer method was used to adjust *p*-values from multiple pairwise comparisons of inputs θ, 2IMU, 2IMU + θ, 4IMU, and 4IMU + θ. The significance threshold for statistical testing was set to α_i_ = 0.05 but was adjusted following multiple comparisons to avoid Type I error. All statistical tests were performed using the Statsmodels module in Python programming language version 3.10.11.

## 3. Results

**Figure 4:**
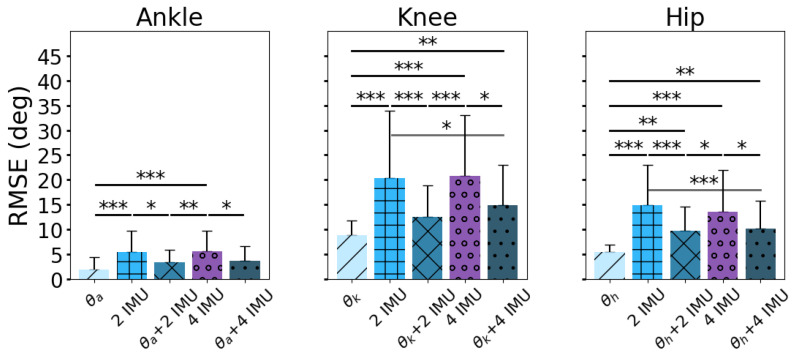
Prediction of RMSE of joint angles 100 ms into the future, showing significant results denoted as * *p* < 0.05, ** *p* < 0.01, and *** *p* < 0.001.

The RMSE values comparing the predictions of the Random Forest algorithm based on altering the sensor feature inputs are shown in Figure 4, where θ_a_, θ_k_, and θ_h_ represent the joint angles of the ankle, knee, and hip, respectively. We ran three independent ANOVAs (one each for the ankle, knee, and hip) and reported degrees of freedom (df), sum of squares (SS), and mean sum of squares (MS), with corresponding F-statistics and *p*-values. The ANOVA tests showed that the main effect of sensor combinations was statistically significant for algorithm prediction at the ankle (F4,279 = 11.07, *p* < 0.001), knee (F4,279 = 15.69, *p* < 0.01), and hip (F4,279 = 18.92, *p* < 0.001), prompting post hoc Tukey HSD pairwise comparisons (Table 1). Tukey HSD pairwise comparisons showed significant differences (*p* < 0.05) in RMSE values (Table 2, Table 3 and Table 4). Table 2, Table 3 and Table 4 show Tukey HSD pairwise comparisons for the ankle, knee, and hip to highlight how different sensor configurations led to more accurate predictions for specific joint angles. Pairwise comparisons resulted in the mean difference in RMSE values between sensor conditions, an adjusted *p*-value (*p*-adj) for each comparison, lower and upper bounds of the 95% confidence interval for the mean difference, and Cohen’s *d,* which shows the effect size as the difference between sensor conditions. The prediction of RMSE (mean ± one standard deviation) for one input (current joint angle, θ) resulted in the lowest predicted RMSE values of 1.92 ± 2.48°, 8.78 ± 2.96°, and 5.48 ± 1.47° for the ankle, knee, and hip, respectively (Figure 4). Two IMUs resulted in predicted RMSE values of 5.35 ± 4.28°, 20.29 ± 13.71°, and 14.86 ± 8.17° for the ankle, knee, and hip, which were not significantly different from the four IMU inputs to predict ankle (5.55 ± 4.10°, *p* < 0.99), knee (20.71 ± 12.41°, *p* < 0.99), and hip angles (13.55 ± 8.41°, *p* < 0.80; Table 2, Table 3 and Table 4).

**Figure 5:**
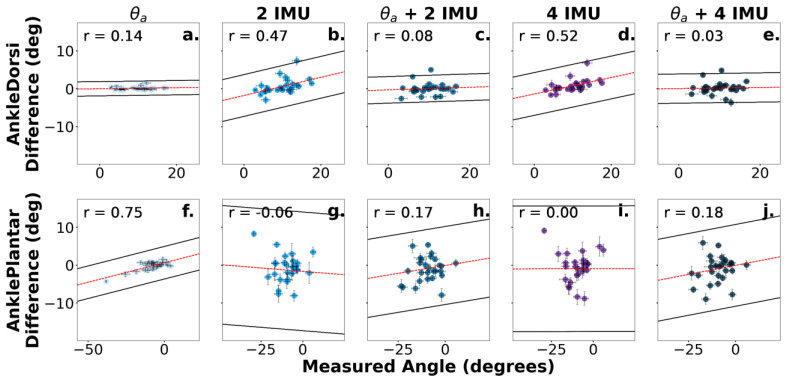
Bland–Altman plots corresponding to the difference between the predicted and measured ankle angles. Sensor combinations are displayed for ankle dorsiflexion (**a**–**e**) and ankle plantarflexion (**f**–**j**). The dotted red line represents the slope of the differences between the predicted and measured angles. The scatterplot colors are darkened for additional number sensor inputs and purple is shown for 4 IMUs. The 95% prediction limits and Pearson correlation coefficients assessing the proportional bias are displayed in the upper left corner of each plot. Error bars represent the standard error.

**Figure 6:**
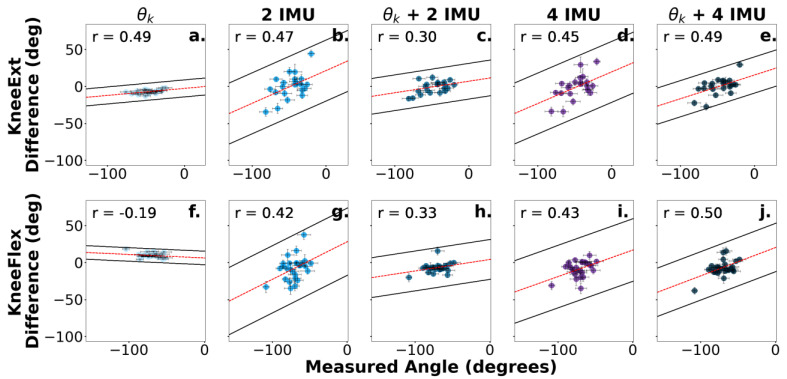
Bland–Altman plots corresponding to the difference between the predicted and measured knee angles. Sensor combinations are displayed for ankle dorsiflexion (**a**–**e**) and ankle plantarflexion (**f**–**j**). The dotted red line represents the slope of the differences between the predicted and measured angles. The scatterplot colors are darkened for additional number sensor inputs and purple is shown for 4 IMUs. The 95% prediction limits and Pearson correlation coefficients assessing the proportional bias are displayed in the upper left corner of each plot. Error bars represent the standard error.

**Figure 7:**
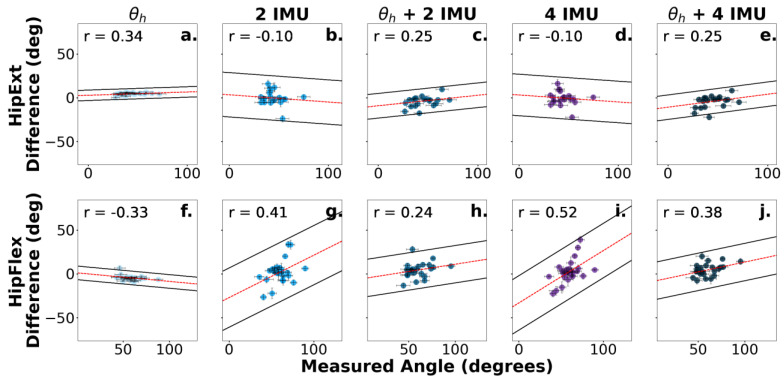
Bland–Altman plots corresponding to the difference between the predicted and measured hip angles. Sensor combinations are displayed for ankle dorsiflexion (**a**–**e**) and ankle plantarflexion (**f**–**j**). The dotted red line represents the slope of the differences between the predicted and measured angles. The scatterplot colors are darkened for additional number sensor inputs and purple is shown for 4 IMUs. The 95% prediction limits and Pearson correlation coefficients assessing the proportional bias are displayed in the upper left corner of each plot. Error bars represent the standard error.

The qualitative assessments of the Bland–Altman plots showed that as the measured angle of the joint increased, the difference between the predicted and measured angles also increased (Figure 5, Figure 6 and Figure 7). 

## 4. Discussion

The purpose of this study was to evaluate various combinations of input signals that maximize machine learning prediction accuracy for simple movements while minimizing the required number of sensors. Our initial hypothesis that prediction accuracy would increase with the addition of IMUs attached to adjacent body segments was rejected. In fact, the prediction RMSE (mean ± one standard deviation) for one input (current joint angle, θ) resulted in the lowest predicted RMSE values of 1.92 ± 2.48°, 8.78 ± 2.96°, and 5.48 ± 1.47° for the ankle, knee, and hip, respectively (Figure 4). In essence, the addition of IMUs neighboring the joint of interest did not improve the joint angle predictions compared to joint angle inputs alone. In other words, starting with only joint angle inputs, incorporating two IMU sensors adjacent to the joint segment into the Random Forest predictor increased the RMSE at the ankle, knee, and hip by an average of 1.40°, 3.76°, and 5.19°, respectively. One plausible explanation for this discrepancy may come from the fact that adjacent IMUs alone (i.e., the two IMU groups) exhibited higher RMSE values compared to joint angle inputs alone (i.e., θ_a_, θ_k_, and θ_h_ groups, as shown in Figure 4). Therefore, adding adjacent IMUs to the joint angle inputs (θ vs. θ + two IMU groups) introduced new uncertainty in the estimates, resulting in a statistically significant worse accuracy at the ankle (RMSE of 1.92 ± 2.48° vs. 3.32 ± 2.59°), knee (RMSE of 8.78 ± 2.96° vs. 12.54 ± 6.28°), and hip (RMSE of 5.48 ± 1.47° vs. 9.67 ± 4.93°). 

Our second hypothesis that the addition of IMUs attached to non-adjacent body segments would not increase prediction accuracy was confirmed because there was not a statistically significant difference between two IMUs vs. four IMUs to predict ankle (5.35 ± 4.28 vs. 5.55 ± 4.10°, *p* < 0.99), knee (20.29 ± 13.71 vs. 20.71 ± 12.41°, *p* < 0.99), and hip angles (14.86 ± 8.17° vs. 13.55 ± 8.41°, *p* < 0.80; Table 2, Table 3 and Table 4). Furthermore, adding non-adjacent segment IMUs to joint angle inputs (four IMUs + θ vs. two IMUs + θ) did not significantly reduce the RMSE values for the ankle, knee, and hip (Table 2, Table 3 and Table 4). This means that the increase in the number of IMU inputs to the Random Forest algorithm from 12 input signals (two IMU sensors each with 3D acceleration and 3D angular velocity per IMU sensor) to 24 input signals (four IMU sensors), or 13 input signals (two IMUs + θ) to 25 input signals (four IMUs + θ), did not reduce RMSE when predicting joint angles. This result suggests that using two IMUs on the adjacent segments of the joint is preferred over four IMUs on the torso, thigh, shank, and foot when predicting ankle, knee, and hip angles. These results align with the notion that classification performance significantly improves when sensors are positioned directly on the body region engaged in the specific movement of interest [40]. Since the IMUs were evaluated on segments exhibiting static and dynamic motion, there is a combination of uncertainty in the initial velocity from raw IMU signals. Furthermore, non-adjacent IMUs (e.g., torso) exhibiting quasi-static behavior may impose a greater amount of uncertainty for predicting dynamic motions (e.g., knee flexion) [41]. In optimal control and estimation theory, the quality of the estimate depends on the reliability and consistency of the sensor data [42]. In this study, uncertainties likely propagated through the estimation algorithm whenever new sources of noise and uncertainty from the IMUs were introduced, leading to increased estimation errors [41]. 

Perhaps the inclusion of four IMU sensors did not enhance the prediction accuracy compared to the two-IMU-sensor configuration because the two additional IMU sensors were not placed proximal and distal to the neighboring joint. This result is noteworthy, as it is more practical to setup two sensors as opposed to four sensors, and removing the number of attached sensors would likely be more convenient for the user while still maintaining a comparable prediction accuracy (Figure 4). The results also agreed with prior work showing how even a single IMU can produce a reasonable classification accuracy for certain manual material handling (MMH) tasks [30]. However, limiting the sensor configuration to a single IMU sensor should be used with caution, as accuracy can substantially decrease if the sensor momentarily drops the signal connection. Also, Porta et al. (2021) showed that prediction accuracy from a single sensor depends on certain MMH tasks, and additional sensors may be needed for tasks where multiple sensors can enhance the prediction accuracy (e.g., push/pull tasks) [30,31]. We also speculated that the addition of IMU sensors did not improve the prediction accuracy because the IMU sensors were not feature-engineered to predict future joint angles. The algorithm predictions, therefore, lagged behind the motion of the limb, as evident by the increased differences as a function of measured joint angles from the Bland–Altman plots (Figure 5, Figure 6 and Figure 7). The Bland–Altman plots showed positive Pearson correlations for 24 out of 30 scenarios, suggesting a general trend of increased differences as the measured joint angle increased (Figure 5, Figure 6 and Figure 7). With the exception of Figure 5f,g,i, and Figure 7b,d,f, the Pearson correlation coefficient revealed that the difference between the Random Forest prediction and the measured joint angle increased as the measured joint angle increased. The Bland–Altman plots also showed that these values were within the 95% limits of agreement, suggesting that the errors were consistently biased in the direction of the measured slope. Cohen’s *d* scores also showed large effect sizes (*d* ≥ 0.8) for a single joint angle input (θ) when compared to IMU inputs for 11 out of 12 comparisons. The only scenario where a large effect did not occur for a single joint angle input (θ) was at the knee (θ_k_ vs. two IMUs + θ_k_), which resulted in a medium effect (*d* = −0.77).

The result of adding relevant types of input sensors to improve the prediction accuracy was in line with prior research [43,44]. For instance, Nurse et al. (2023) showed how the combination of pressure insole data with trunk motion increased low back disorder risk estimates from a range of *r* = 0.20–0.56 to *r* = 0.93–0.98 [43]. Substituting trunk IMU with thigh or pelvis IMU did not significantly reduce the prediction accuracy; however, removing more relevant sensors, such as force estimates from pressure insoles, significantly reduced low-back-loading estimation accuracy during MMH [45]. Smartphone camera data have also been shown to improve the prediction accuracy of knee adduction moment and knee flexion moment when combined with IMU sensors compared to models that used IMU or cameras alone [46]. Furthermore, optimizing the sensor configuration for HMIP for lower-limb joints aligns with previous research that validated the accurate prediction of ankle, knee, and hip angles [47]. This offers potential for the practical application of industrial exoskeletons during lifting activity [47]. However, the success of predictors not only depends on application but also on algorithm development and may still require sophisticated deep learning algorithms, such as LSTM, to obtain accurate estimates [30,48]. Nevertheless, this study points to implementing strategies for real-world applications where the tradeoff between the number of sensors and prediction accuracy is essential. Such applications include predicting ankle, knee, and hip mobility during rehabilitation, where attaching sensors can be time-consuming and inconvenient for users. Patients recovering from injury may require precise monitoring of joint angles across simple movements, and this study showed how a single sensor measuring joint angles can accurately predict ankle, knee, and hip angles, with predicted RMSE values of 1.92 ± 2.48°, 8.78 ± 2.96°, and 5.48 ± 1.47°. These RMSE values are a small percentage of the typical sagittal plane range of motion for the healthy adult population at the ankle (70–80°), (55–60°), knee (140–155°), and hip (130°) [49].

It is also worth noting that machine learning algorithms trained with a single feature input of joint angles resulted in statistically lower predicted RMSE value compared to algorithms with multiple IMUs along with joint angle inputs (Table 2, Table 3 and Table 4). It is possible that this result occurred because the IMU signals were not feature-engineered. Although the input signals were normalized, we did not perform other feature engineering techniques to extract relevant information. Additional feature extraction techniques that could have enhanced prediction accuracy using IMU sensor inputs include sensor fusion, strapdown integration of the gyroscope signal to acquire displacement, and statistical features across a moving time window, such as mean, minimum, maximum, standard deviation, and kurtosis [21]. Due to the complexities and limitations of feature engineering techniques, such as drift error, this study did not perform feature engineering techniques, apart from normalization. More complex deep learning models, such as artificial neural networks, autoencoders, and long short-term memory, are capable of extracting relevant features from raw data and may have an improved prediction accuracy with the addition of adjacent raw IMU inputs. However, it is important to consider factors such as model complexity, runtime, and hyperparameter tuning when implementing complex deep learning models.

This study consisted of some limitations. First, the recorded activities were simple movements performed in isolation. The study did not evaluate the algorithm during multi-joint movement tasks, nor was it tested during transitions between tasks. Activities of daily living are rarely performed in isolation, as human motion is a sequence of one action to another. Since the Random Forest algorithm was not trained with the transition from one task to another, it may incur prediction errors when predicting joint angles between tasks. As a result, the algorithm may be incapable of predicting multiple actions in a fluid-like manner. Therefore, future studies should include more complex actions and even transitions between actions in the training data to avoid overfitting to isolated tasks. Nonetheless, we believe this study serves as an initial baseline for future studies to effectively predict joint angles during complex movements. Another limitation was that this was an offline analysis. Future work should consider how runtime may alter prediction accuracy in an online scenario, where delays are critical for predicting user intent. Finally, this study was only performed on a healthy young adult population. As a result, additional data collection from diverse user populations, such as the elderly or amputees, is recommended for HMIP tailored to a specific population. Additionally, a mixture of training data from multiple populations could enhance the overall generalizability of the machine learning model, which can be used for activities that were not performed in this study, such as fall detection and running [50,51,52,53].

In order to offset the prediction bias, the Random Forest algorithm may benefit from additional sensor inputs, such as EMG signals. Since EMG signals occur approximately 120 ms prior to limb motion, this input information may help combat the lagging behavior of the Random Forest algorithm. As shown by Coker et al. (2020), including joint angles with EMG inputs resulted in RMSE 2.04° for predicting knee flexion angles 100 ms into the future [2]. Furthermore, Hollinger et. al. (2023) used EMG signals in addition to IMU and kinematic inputs for future joint angle prediction, which did not result in a prediction bias during level-ground walking [22]. However, the predictive algorithms by Hollinger et al. (2023) and Coker et al. (2020) were limited to level-ground walking. Therefore, EMG signals, feature engineering of IMUs, or a combination of both may help with joint angle prediction across diverse actions and may be worth exploring in future studies. Although this study focused on the growing topic of machine learning for HMIP, the applications of HMIP have a wide range of potential applications in monitoring rehabilitation, controlling a wearable exoskeleton, and identifying risk factors for work-related musculoskeletal disorders [44].

## 5. Conclusions

The results from this study indicated that machine learning prediction accuracy did not necessarily improve with additional IMU sensors. For instance, the addition of non-adjacent IMUs compared to two IMU sensors attached to adjacent segments did not increase the accuracy of predicting ankle, knee, and hip angles. This study also showed that using only kinematic inputs (joint angles) resulted in the best prediction accuracy of the Random Forest algorithm for the ankle, knee, and hip. This study, therefore, demonstrated how future joint angle prediction is largely determined by previous joint angles and is the most practical input signal for maximizing the joint angle prediction accuracy for multiple simple movements.

## Figures and Tables

**Table 1:** Analysis of variance results for the ankle, knee, and hip. A bold *p*-value indicates a statistically significant result (*p* < 0.05).

Source	df	SS	MS	F	*p*
Ankle between groups	4	3921.21	980.30		
Ankle within groups	51	4069.78	79.79		
Ankle total	279	7991.00	None	11.07	**<0.001**
Knee between groups	4	57,979.06	14,494.76		
Knee within groups	51	38,835.67	761.48		
Knee total	279	96,814.73	None	15.69	**<0.001**
Hip between groups	4	28,201.66	7050.41		
Hip within groups	51	17,439.06	341.94		
Hip total	279	45,640.7	None	18.92	**<0.001**

**Table 2:** Post hoc pairwise Tukey’s honest significant difference—ankle condition.

Group 1	Group 2	Mean Diff.	*p*-adj.	Lower	Upper	Cohen’s *d*
2 IMU	2 IMU + θ_a_	−2.03	<0.01	−3.80	−0.27	−0.57
2 IMU	4 IMU	0.20	0.99	−1.57	1.96	0.05
2 IMU	4 IMU + θ_a_	−1.69	0.070	−3.46	0.070	−0.46
2 IMU	θ_a_	−3.43	<0.001	−5.20	−1.67	−0.98
2 IMU + θ_a_	4 IMU	2.23	<0.01	0.47	4.00	0.65
2 IMU + θ_a_	4 IMU + θ_a_	0.34	0.98	−1.42	2.10	0.12
2 IMU + θ_a_	θ_a_	−1.40	0.19	−3.16	0.36	−0.55
4 IMU	4 IMU + θ_a_	−1.89	0.029	−3.66	−0.13	−0.53
4 IMU	θ_a_	−3.63	<0.01	−5.40	−1.87	−1.07
4 IMU + θ_a_	θ_a_	−1.74	0.055	−3.50	0.024	−0.64

**Table 3:** Post hoc pairwise Tukey’s honest significant difference—knee condition.

Group 1	Group 2	Mean Diff.	*p*-adj.	Lower	Upper	Cohen’s *d*
2 IMU	2 IMU + θ_k_	−7.76	<0.001	−12.76	−2.74	−0.73
2 IMU	4 IMU	0.41	0.99	−4.59	5.42	0.03
2 IMU	4 IMU + θ_k_	−5.48	0.023	−10.49	−0.47	−0.49
2 IMU	θ_k_	−11.51	<0.001	−16.52	−6.50	−1.16
2 IMU + θ_k_	4 IMU	8.17	<0.001	3.16	13.18	0.83
2 IMU + θ_k_	4 IMU + θ_k_	2.27	0.72	−2.73	7.28	0.31
2 IMU + θ_k_	θ_k_	−3.75	0.24	−8.76	1.25	−0.77
4 IMU	4 IMU + θ_k_	−5.90	0.011	−10.91	−0.89	−0.56
4 IMU	θ_k_	−11.92	<0.001	−16.93	−6.91	−1.32
4 IMU + θ_k_	θ_k_	−6.02	<0.01	−11.03	−1.01	−0.98

**Table 4:** Post hoc pairwise Tukey’s honest significant difference—hip condition.

Group 1	Group 2	Mean Diff.	*p*-adj.	Lower	Upper	Cohen’s *d*
2 IMU	2 IMU + θ_h_	−5.19	<0.001	−8.47	−1.90	−0.77
2 IMU	4 IMU	−1.31	0.80	−4.59	1.97	−0.16
2 IMU	4 IMU + θ_h_	−4.79	<0.001	−8.07	−1.50	−0.68
2 IMU	θ_h_	−9.38	<0.001	−12.66	−6.09	1.60
2 IMU + θ_h_	4 IMU	3.87	0.011	0.59	7.16	0.56
2 IMU + θ_h_	4 IMU + θ_h_	0.39	0.99	−2.88	3.68	0.07
2 IMU + θ_h_	θ_h_	−4.19	<0.01	−7.47	−0.90	1.15
4 IMU	4 IMU + θ_h_	−3.47	0.031	−6.76	−0.19	−0.48
4 IMU	θ_h_	−8.07	<0.001	−11.35	−4.78	1.34
4 IMU + θ_h_	θ_h_	−4.59	<0.01	−7.87	−1.30	1.10

## Data Availability

The raw data supporting the conclusions of this article will be made available by the authors upon request.
